# Hyperglycemia in Severe and Critical COVID-19 Patients: Risk Factors and Outcomes

**DOI:** 10.7759/cureus.27611

**Published:** 2022-08-02

**Authors:** Viet Tran Le, Quoc Hung Ha, Minh Triet Tran, Ngoc Trong Le, Van Tuyen Le, Minh Khoi Le

**Affiliations:** 1 COVID-19 Intensive Care Center, University Medical Center, Ho Chi Minh City, VNM; 2 Department of Endocrinology, University Medical Center, Ho Chi Minh City, VNM; 3 Critical Care Medicine, University of Medicine and Pharmacy at Ho Chi Minh City, Ho Chi Minh City, VNM

**Keywords:** corticosteroid, intensive care, diabetes, hyperglycemia, covid-19

## Abstract

Background: Hyperglycemia is commonly seen in critically ill patients. This disorder was also seen in coronavirus disease 2019 (COVID-19) patients and was associated with a worse prognosis. The current study determined the prevalence, risk factors, and prognostic implications of hyperglycemia in COVID-19 patients.

Method: This was a retrospective observational study performed in an intensive care unit for COVID-19 patients. Electronic data of COVID-19 patients admitted to the intensive care unit from August 2nd to October 15th, 2021, were collected. Patients were divided into non-hyperglycemia, hyperglycemia in diabetic patients, and hyperglycemia in non-diabetic patients. Primary outcomes were 28-day and in-hospital mortalities. Multinomial logistic regression and multivariable Cox regression models were used to determine the risk factors for hyperglycemia and mortality, respectively.

Results: Hyperglycemia was documented in 65.6% of patients: diabetic patients (44.8%) and new-onset hyperglycemia (20.8%). In-hospital and 28-day mortality rates were 30.2% and 26.1%, respectively. Respiratory failure, corticosteroid therapy, and a higher level of procalcitonin were risk factors for hyperglycemia in diabetic patients, whereas cardiovascular diseases, respiratory failure, and higher aspartate aminotransferase/glutamate aminotransferase ratio were risk factors for hyperglycemia in non-diabetic patients. The risk of the 28-day mortality rate was highest in the new-onset hyperglycemia (hazard ratio [HR] 3.535, 95% confidence interval [CI] 1.338-9.338, p=0.011), which was higher than hyperglycemia in type 2 diabetes mellitus patients (HR 1.408, 95% CI 0.513-3.862, p=0.506).

Conclusion: Hyperglycemia was common in COVID-19 patients in the intensive care unit. Hyperglycemia reflected the disease severity but was also secondary to therapeutic intervention. New-onset hyperglycemia was associated with poorer outcomes than that in diabetic patients.

## Introduction

Since December 2019, the severe acute respiratory syndrome coronavirus 2 (SARS-CoV-2) causing coronavirus disease 2019 (COVID-19) has spread rapidly across the globe and claimed more than 511 million infected cases and 6.22 million deaths, making it one of the deadliest plagues in the human history [1].

Chronic comorbidities are common in severe and critical COVID-19 patients [2]. Type 2 diabetes mellitus (T2DM) was the second most frequent chronic disease in COVID-19 patients and was a risk factor for severity and mortality in this group of patients [3-5]. Compared to baseline hemoglobin A1C (HbA1C), acute hyperglycemia in diabetes is a stronger predictor of death in critically ill diabetic patients admitted to intensive care units (ICU) [6,7]. Hyperglycemia is also common among non-diabetic patients in ICU, and the more severe hyperglycemia is, the higher the risk of respiratory failure, infection, and mortality [8,9]. Interestingly, acute hyperglycemia is more harmful to non-diabetic patients than to diabetic patients in the ICU [10]. It was demonstrated that diabetes and hyperglycemia were the risk factors for a poorer prognosis of the previous severe acute respiratory syndrome (SARS) disease [11]. Early in the COVID-19 pandemic, it was recognized that pre-existing T2DM, newly diagnosed T2DM, and new-onset hyperglycemia were associated with a worse prognosis in COVID-19 patients [12,13]. 

Hyperglycemia in critically ill patients is caused by multiple pathological conditions, including inflammatory reactions. Cytokine storm plays a central role in the pathology of COVID-19 [14]. It is not surprising that hyperglycemia occurs in COVID-19 patients [5]. However, different from other critical diseases and also different from those in the early phase of the COVID-pandemic, the wide adoption of corticosteroid therapy (CST), especially mini pulse CST in the second half of the year 2021, might affect the epidemiology and the outcome of acute hyperglycemia in COVID-19 patients. This study was performed in this clinical context to investigate the incidence, the possible risk factors of hyperglycemia, and the effect of hyperglycemia in 28-day in-hospital mortality in COVID-19 patients.

## Materials and methods

Study design

This retrospective observational study recruited all COVID-19 patients, confirmed with positive reverse transcription polymerase chain reaction (RT-PCR) for SARS-CoV-2, admitted to our COVID-19 intensive care center from August 2nd to October 15th, 2021. The diagnosis of COVID-19 was based on a positive RT-PCR for SARS-CoV-2 following the World Health Organization (WHO) interim guidance [15]. We excluded patients with type 1 diabetes mellitus, pregnancy, patients under 18 years of age, patients in whom a presence of a T2DM was not confirmed or excluded with certainty, and patients whose electronic medical record was not well documented. This center, a part of a field hospital, was a tertiary referral center established during the peak of the COVID-19 pandemic that ravaged the city of 10 million inhabitants.

Data collection

The relevant data were extracted from the electronic medical records. Data of each patient were collected by two investigators. Any collected information that required further clarification was reviewed by the most senior investigators or by the whole team.

We mostly used capillary blood glucose (BG) values in clinical practice. The venous BG from the central laboratory was issued on a daily checkup or on demand, usually in combination with other hematological, biochemical, and immunological tests. Should the venous BG report an abnormal result, we used the capillary BG for closer monitoring. This study used the capillary BG values for analysis. All patients had their capillary BG values measured on admission to the ICU.

Hyperglycemia was defined when a patient had at least two random BG values >180 mg/dL in 24 hours. Based on the BG level during the hospital stay and history of T2DM, we divided the patients into three groups as follows:

Non-hyperglycemia: non-diabetic or T2DM patients who did not meet the criteria of hyperglycemia during their hospital stay;

Hyperglycemia with T2DM: at least two random BG values >180 mg/dL in 24 hours and the patient had been previously diagnosed with T2DM; and

New-onset hyperglycemia: at least two random BG values >180 mg/dL in 24 hours and the T2DM was excluded.

The primary outcome was 28-day and in-hospital mortality in the three groups mentioned above.

Statistical analysis

The statistical analysis was performed using the SPSS Statistics, version 28.0.1.0 (IBM, Armonk, NY, USA). Data are expressed as frequency (percentage) for categorical variables; mean ± standard deviation, and median (interquartile range) for continuous parameters. The one-way analysis of variance (ANOVA) was used to compare three groups. In univariate analysis, the chi-square test was used for categorical parameters and the Wilcoxon rank-sum test for continuous parameters to compare the survival and death groups. The variables that had significance in the univariate analysis were included in multivariate Cox (proportional-hazards) regression to identify the independent risk factors of in-hospital mortality. All statistical tests were two-tailed, and a p-value of less than 0.05 was considered statistically significant.

Research ethics

The present study was approved by the Institutional Review Board of University Medical Center, Ho Chi Minh City, Vietnam (approval number: 08022022/HDDD-BVDHYD). The informed consent for participation was obtained from the patients or their family members. All methods were performed in accordance with the Declaration of Helsinki.

## Results

Demographic, laboratory, and therapeutic characteristics

After screening, 517 patients who met the inclusion criteria were recruited for the study. The demographic and clinical characteristics of the three groups on admission are presented in Table 1. The incidence of hyperglycemia was 65.6%. The non-hyperglycemia group was younger than the two groups with hyperglycemia. The two groups with hyperglycemia were comparable in age and body mass index (BMI), but the T2DM hyperglycemia group had a significantly higher percentage of female patients (62.9% vs. 52.3%). Comorbidities were common in three groups, with arterial hypertension more dominant in the T2DM hyperglycemia group. On admission, clinical parameters were not significantly different between the two groups with hyperglycemia. The respiratory failure in the non-hyperglycemia was less severe than in the rest. In the non-hyperglycemia group, there were 24 diabetic patients (13.5%). The new-onset hyperglycemia incidence in non-diabetic patients was 41.0% (107/261 patients).

**Table 1:** Characteristics of COVID-19 patients in three groups based on glycemic status. Data are expressed as frequency (percentage) for categorical variables; mean ± standard deviation, and median (interquartile range) for continuous parameters. (*) Dexamethasone or equivalent. T2DM: type 2 diabetes mellitus; BG: blood glucose; BMI: body mass index; CKD: chronic kidney disease; CS: corticosteroid; CST: corticosteroid therapy; CV: cardiovascular; FiO_2_: fraction of inspired oxygen; HFNC: high-flow nasal cannula; IMV: invasive mechanical ventilation; NIV: non-invasive ventilation; P/F: PaO_2_/FiO_2_; SBP: systolic blood pressure; SpO_2_: peripheral oxygen saturation.

Parameters	Non-hyperglycemia (n = 178, 34.4%)	T2DM hyperglycemia (n = 232, 44.8%)	New-onset hyperglycemia (n = 107, 20.8%)	p-Value
Demographic characteristics				
Male:female	94:84	86:146	51:56	0.005
Age (years)	57.3 ± 12.5	65.2 ± 13.7	64.1 ± 15.7	<0.001
BMI (kg/m^2^)	23.4 ± 3.6	24.4 ± 4.1	23.8 ± 4.1	0.073
Vaccinated n (%)	26 (14.6)	19 (8.2)	9 (8.4)	0.081
28-day mortality n (%)	7(3.9)	80 (34.5)	48 (44.9)	<0.001
In-hospital mortality n (%)	8 (4.5)	94 (40.5)	54 (50.5)	<0.001
Chronic comorbidities				
T2DM	24 (13.5)	232 (100.0%)	0 (0%)	<0.001
Hypertension n (%)	78 (43.8)	165 (71.1)	53 (49.5)	<0.001
CV disease n (%)	18 (10.1)	35 (15.1)	21 (19.6)	0.077
Pulmonary disease n (%)	15 (8.4)	13 (5.6)	9 (8.4)	0.466
CKD n (%)	9 (5.1)	27 (11.6)	7 (6.5)	0.043
Hepatic disease n (%)	6 (3.3)	8 (3.5)	5 (5.6)	0.580
Vital signs on admission				
Heart rate (bpm)	91.8 ± 17.9	95.6 ± 18.6	93.8 ± 17.5	0.124
SBP (mmHg)	125.2 ± 20.0	128.6 ± 23.7	125.4 ± 26.3	0.263
Respiratory rate (bpm)	22 (20-25)	25 (22-30)	25 (22-30)	<0.001
Temperature (^o^C)	37 (37-37)	37 (37-37)	37 (37-37)	0.649
SpO_2_ (%)	95 (97-91)	92 (84-96)	92 (85.5-96.0)	<0.001
P/F ratio on admission	161 (116-274)	131 (91-219)	111 (83-183)	<0.001
Respiratory support on admission				
On room air n (%)	23 (12.9)	6 (2.6)	1 (0.9)	<0.001
Nasal cannula n (%)	49 (27.5)	43 (18.5)	13 (12.2)	0.005
Non-rebreathing face mask n (%)	38 (21.3)	29 (12.5)	12 (11.2)	0.020
HFNC n (%)	58 (32.6)	94 (40.5)	48 (44.9)	0.089
NIV n (%)	0 (0.0)	6 (2.6)	0 (0.0)	0.029
IMV n (%)	10 (5.6)	54 (23.3)	33 (30.8)	<0.001
Corticosteroid therapy				
Total CS dose (mg)*	85.8 (52.8-131.3)	99 (66.0-135.8)	102.8 (69.3-151.8)	0.089
Average CS dose (mg/day)*	8.1 (6.6-11.3)	9.6 (6.6-11.6)	9.9 (6.6-12.6)	0.020
CST duration (days)	10 (7-11)	10 (8-12)	10 (7-12)	0.379
Mini-pulse CST n (%)	32 (18.0)	44 (19.0)	24 (22.4)	0.642

As well recognized as a factor inducing hyperglycemia, CST was also investigated in the current study. Briefly, the new-onset hyperglycemia group seemed to receive slightly higher doses of corticosteroids than the two other groups.

The laboratory results on the admission of the three groups of patients are presented in Table 2. Both T2DM and new-onset hyperglycemia groups demonstrated higher levels of inflammatory markers, namely white blood cell counts (WBC), C-reactive protein (CRP), D-dimer, and interleukin-6, except for the plasma fibrinogen concentration. The procalcitonin concentrations of the three groups were statistically different in absolute values as well as in terms of the proportions of patients with procalcitonin levels equal to or above 0.5 ng/mL. The renal function of T2DM hyperglycemia and new-onset hyperglycemia groups was more depressed than the non-hyperglycemia group. Liver function tests in three groups showed a mild degree of hepatic damage with a higher aspartate aminotransferase/glutamate aminotransferase (AST/ALT) ratio in the new-onset hyperglycemia group.

**Table 2:** Laboratory features of COVID-19 patients in three groups based on glycemic status. Data are expressed as frequency (percentage) for categorical variables; mean ± standard deviation, and median (interquartile range) for continuous parameters. BG: blood glucose; CRP: C-reactive protein; PCT: pro-calcitonin; AST: aspartate aminotransferase; ALT: glutamate aminotransferase; eGFR: estimated glomerular filtration rate; HbA1C: hemoglobin A1C; WBC: white blood cell counts.

Parameters	Non- hyperglycemia (n = 178)	T2DM hyperglycemia (n = 232)	New-onset hyperglycemia (n = 107)	p-Value
Blood glucose level				
BG on admission (mg/dL)	141.5 (119-174.8)	255.5 (175-375.8)	160 (134-201)	0.000
Average BG level (mg/dL)	137.7 (123.4-153.0)	224.9 (198.6-268.0)	177.4 (144-214.0)	0.000
HbA1C (%)	6.1 (5.8-6.4)	7.4 (6.8-9.2)	6.0 (5.8-6.3)	0.000
Admission laboratory features				
WBC (g/L)	8.7 (6.4-12.4)	9.8 (7.3-13.5)	9.7 (6.3-12.9)	0.075
CRP (mg/L)	49.8 (18.6-100.5)	57.9 (27.0-123.5)	72.7 (35.5-110.0)	0.030
Ferritin (ng/mL)	1128 (663-1680)	1139 (684-1675)	1112 (788-1676)	0.922
D-dimer (ng/mL)	880 (524-2020)	1261 (675-654)	1540 (831-2915)	<0.001
Interleukin-6 (pg/mL)	15.8 (7.7-38.2)	25.6 (13.2-67.2)	20.1 (11.1-40.5)	0.001
Fibrinogen (g/L)	5.1 ± 1.4	5.1 ± 1.3	4.9 ± 1.4	0.616
PCT (ng/mL)	0.130 (0.100-0.295)	0.271 (0.100-0.800)	0.200 (0.100-0.840)	<0.001
PCT ≥ 0.5 ng/mL (%)	31 (17.4)	86 (37.1)	39 (36.5)	<0.001
Creatinine (μmol/L)	75.1 ± 27.6	87.4 ± 34.7	85.3 ± 35.3	<0.001
eGFR (mL/min/1.73 m^2^)	86 (69-115)	69 (47-92)	78 (54-100)	<0.001
AST (IU/L)	50 (34.3-96.8)	47.5 (30.3-76.8)	70 (43.5-102)	<0.001
ALT (IU/L)	54.5 (28-106.8)	41 (26-66)	48 (32-84)	0.007
AST/ALT ratio	1.0 (0.7-1.7)	1.2 (0.8-1.6)	1.5 (0.7-2.1)	<0.001

Risk factors of T2DM hyperglycemia and new-onset hyperglycemia

We further investigated the independent risk factors of T2DM hyperglycemia and new-onset hyperglycemia. The analysis results are presented in Table 3. The invasive mechanical ventilation on admission, procalcitonin level of more than 0.5 ng/mL, and CST were the risk factors for hyperglycemia in T2DM patients. In non-diabetic patients, invasive mechanical ventilation on admission was still an important independent risk factor for hyperglycemia. In addition, the presence of cardiovascular diseases and the high AST/ALT ratio were also the risk factors for hyperglycemia in non-diabetic COVID-19 patients.

**Table 3:** Multinomial logistic regression model to determine risk factors of T2DM hyperglycemia and new-onset hyperglycemia. Nagelkerke's pseudo r-squared: 0.733. ALT: alanine aminotransferase; AST: aspartate aminotransferase; CI: confidence interval; CRP: C-reactive protein; CST: corticosteroid therapy; CV: cardiovascular; DXM: dexamethasone (or equivalent); IMV: invasive mechanical ventilation; n.s.: not significant; OR: odds ratio; PCT: procalcitonin; ref.: reference.

	T2DM hyperglycemia			New-onset hyperglycemia		
Covariates	OR	95% CI	p-Value	OR	95% CI	p-Value
Age (years)			n.s.	1.026	1.002-1.049	0.030
Sex			n.s.			n.s.
Hypertension			n.s.			n.s.
CV disease			n.s.	3.704	1.503-9.129	0.004
IMV on admission	3.547	1.226-10.261	0.019	6.073	2.405-15.334	<0.001
PaO_2_/FiO_2_ ratio			n.s.	0.994	0.991-0.998	0.003
D-dimer (ng/mL)			n.s.			n.s.
CRP (mg/dL)			n.s.			n.s.
Creatinine (mg/dL)			n.s.			n.s.
AST/ALT ratio			n.s.	1.523	1.034-1.034	0.033
PCT ≥ 0.5 ng/mL	2.642	1.028-6.793	0.044			n.s.
Corticosteroid therapy						
No CST	1.000		ref.	1.000		ref.
DXM 6.6 mg/day	11.766	1.740-79.576	0.011			n.s.
DXM > 6.6 mg/day	29.558	4.236-206.243	<0.001			n.s.

Patients’ outcomes based on the glycemic status

Out of 517 patients enrolled in the study, 156 (30.2%) patients died in the hospital. The 28-day mortality was 3.9% (7/178), 34.5% (80/232), and 44.9% (48/107) in the non-hyperglycemia, T2DM hyperglycemia, and new-onset hyperglycemia group, respectively.

**Figure 1:**
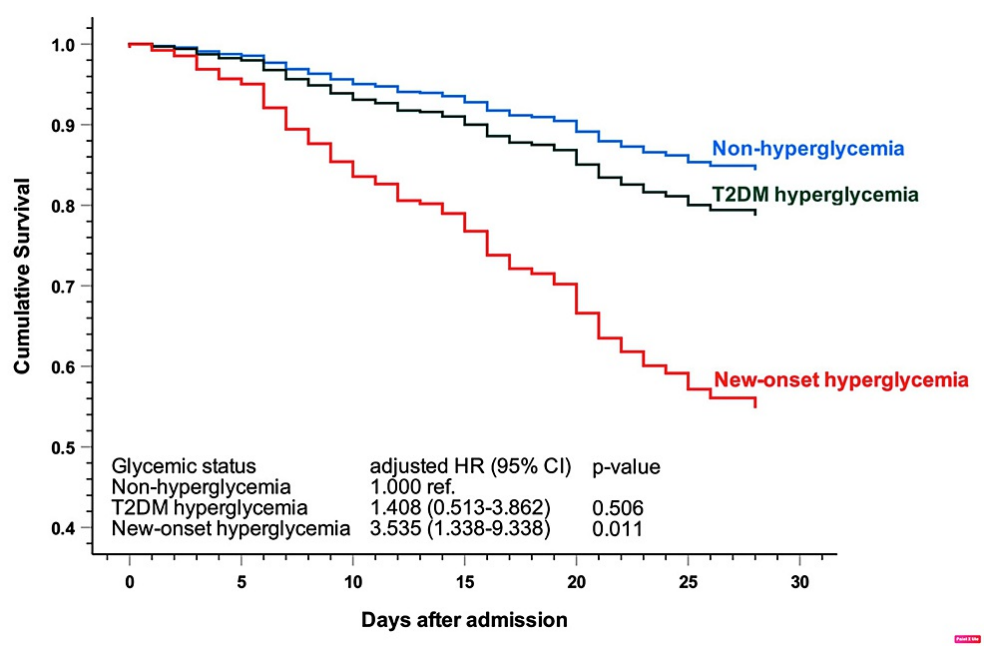
Survival curve from multivariate Cox regression model of different glycemic status. CI: confidence interval; HR: hazard ratio; T2DM: type 2 diabetes mellitus.

The hazard ratio (HR) for death with adjustment for other risk factors was significantly increased among patients with hyperglycemia with or without T2DM compared to those of the non-hyperglycemia group (Figure 1).

We also noticed that the percentage of patients requiring invasive mechanical ventilation was significantly higher (70%) in the new-onset hyperglycemia group than in the T2DM hyperglycemia group (51%) and in patients without hyperglycemia (9%).

Independent predictors of COVID-19 mortality

We also performed a multivariable Cox regression model to identify the risk factors of 28-day in-hospital mortality. The results are presented in Table 4. Out of 13 parameters investigated, higher age, increased mean glucose level, D-dimer, new-onset hyperglycemia, PaO_2_/FiO_2_ ratio on admission, PCT ≥ 0.5 ng/mL, and average daily corticosteroid dose were independently associated with increased risk of mortality. Interestingly, the new-onset hyperglycemia was associated with an important increase in in-hospital mortality (HR 3.535, 95% confidence interval [CI] 1.338-9.338, p = 0.011).

**Table 4:** Risk factors of 28-day in-hospital mortality in patients with COVID-19. Analysis was performed by using a multivariable Cox regression model. AST: aspartate aminotransferase; CI: confidence interval; CRP: C-reactive protein; CS: corticosteroid (dexamethasone equivalent); HR: hazard ratio; IL-6: interleukin-6; n.s.: not significant; PCT: procalcitonin; ref.: reference.

Covariates	HR	95% CI	p-Value
Age (years)	1.046	1.004-1.052	0.023
Sex (male)			n.s.
Hypertension			n.s.
PaO_2_/FiO_2_ ratio	0.997	0.994-0.999	0.007
D-dimer (mg/L)	1.028	1.004-1.052	0.023
CRP (mg/dL)			n.s.
IL-6 (pg/mL)			n.s.
PCT ≥ 0.5 ng/mL	2.254	1.486-3.419	<0.001
Creatinine (mg/dL)			n.s.
AST (IU/L)			n.s.
Average glucose (mg/dL)	1.012	1.009-1.016	<0.001
Average CS dose (mg/day)	1.029	1.005-1.054	0.016
Glycemic status			
Non-hyperglycemia	1.000		ref.
T2DM hyperglycemia	1.408	0.513-3.862	0.506
New-onset hyperglycemia	3.535	1.338-9.338	0.011

## Discussion

It is well established that diabetes significantly increases the risk of developing and dying from infectious diseases [16,17]. Hyperglycemia is a common manifestation directly correlated with increased mortality or morbidity in critically ill patients [18-20]. Early in the COVID-19 pandemic, the first studies found that diabetes was one of the most common comorbidities in COVID-19 patients [4,21]. In COVID-19 patients, hyperglycemia may be further accentuated due to the intense cytokine storm [22]. Little evidence existed on whether hyperglycemia in COVID-19 patients bears any significant prognostic implication. The current study investigated the incidence of hyperglycemia, the risk factors of hyperglycemia in diabetic and non-diabetic COVID-19 patients, and the impact of hyperglycemia on mortality and morbidity.

The incidence of hyperglycemia was noticeably high (65.6%) in the studied population. Hyperglycemia-associated mortality in critically ill patients and the beneficial effects of glycemic control have been intensively studied since the breakthrough work by Van den Berghe et al. [8]. These studies have shed light on the mechanism of hyperglycemia and its harmful consequence in this group of patients. The mechanisms of hyperglycemia in COVID-19 patients are likely multifactorial and were discussed in depth elsewhere [22]. The incidence of hyperglycemia in our study was higher than that in a study by Bode et al., including COVID-19 patients from 88 hospitals in the United States, where hyperglycemia was documented in 40% of patients [23]. Several possible reasons for the higher incidence of hyperglycemia in the current study should be mentioned. Our study was conducted in a field hospital built when the COVID-19 pandemic reached its peak, causing overwhelming in the healthcare facility and human resources. These factors might reduce the required adhesion to protocolized management, including glycemic control in critically ill patients. Furthermore, the current study was performed after nearly two months of strict travel restrictions and city lockdown. The consequent lifestyle change, reduced physical activity, poorly controlled diets, and difficulty in medical access, especially the diabetic medications, might partially explain the high incidence of hyperglycemia [22].

The new-onset hyperglycemia was documented in 20.8% of all patients and 41.0% of non-diabetic COVID-19 patients. This incidence was lower than that (28.4%) in the retrospective study by Li et al. [13]. More importantly, the widespread CST therapy, especially the mini-pulse dose, was obviously responsible for hyperglycemia, as shown in Table 3. Previous studies showed that 53-70% of non-diabetic individuals developed steroid-induced hyperglycemia after being treated with high-dose corticosteroids [24,25].

The 28-day and the in-hospital mortality rates were 26.1% and 30.2%, respectively. Our center was the tertiary referral hospital receiving the most severe COVID-19 patients during the zenith of the pandemic. This might explain the higher mortality rate compared to previous studies from China and United States. Besides a higher 28-day mortality, the new-onset hyperglycemia was also related to an increased need for mechanical ventilation during the hospitalization (70%) compared to the T2DM hyperglycemia group (51%). The current study confirmed once again the findings in previous works [3,26]: the new-onset hyperglycemia was associated with poorer outcomes in COVID-19 patients. Compared to the non-hyperglycemia group, the risk of the 28-day mortality rate was highest in the new-onset hyperglycemia (HR 3.535, 95% CI 1.338-9.338, p = 0.011), which was higher than T2DM hyperglycemia (HR 1.408, 95% CI 0.513-3.862, p = 0.506). The clinical and laboratory findings in T2DM hyperglycemia and new-onset hyperglycemia in the current study indicated the more severe manifestations in these two groups compared to the rest. At the first glance, the severity in the two hyperglycemia groups was not significant. However, the levels of CRP (72.7 mg/L vs. 57.9 mg/L), D-dimer (1540 ng/mL vs. 1139 ng/mL), AST/ALT ratio (1.5 vs. 1.2), and the proportion of patients requiring invasive mechanical ventilation (30.8% vs. 23.3%) on admission were higher in the new-onset than in the T2DM hyperglycemia group. This difference in severity may explain why the new-onset hyperglycemia group had poorer outcomes than the T2DM hyperglycemia group. It has been demonstrated that the relationship between hyperglycemia and COVID-19 is a complex and bidirectional interaction: hormone dysregulations with insulin resistance and the intense cytokine storm in COVID-19 induce hyperglycemia. Hyperglycemia, in turn, adversely affects the host immune response [27], increases inflammatory cytokines [28], and facilitates SARS-CoV-2 replication [29]. More importantly, hyperglycemia worsened the progression of respiratory failure [26]. T2DM has been well proven as one of the most common chronic comorbidities and an important risk factor for poorer outcomes and higher mortality in COVID-19 patients. Our study reinforced the evidence in a new demographic, therapeutic, and socio-economic population. The important finding was that the new-onset hyperglycemia patients were associated with higher mortality and other secondary outcomes compared to T2DM patients who developed hyperglycemia during their hospitalization due to COVID-19. The new-onset hyperglycemia mirrored the severity of COVD-19 disease and adversely affected the clinical course of these patients. Therefore, new-onset hyperglycemia is a strong predictor of severity in COVID-19 patients.

## Conclusions

Hyperglycemia was documented in approximately two-thirds of severe and critical COVID-19 patients admitted to ICU. Hyperglycemia in patients with T2DM was more frequently seen and two-fold higher than the new-onset hyperglycemia. The new-onset hyperglycemia group had the highest 28-day and in-hospital mortality rates, followed by the T2DM hyperglycemia group. The lowest mortality rates were documented in the non-hyperglycemia group. Similarly, the percentage of patients requiring invasive mechanical ventilation was significantly higher in the new-onset hyperglycemia group than in the T2DM hyperglycemia group and in patients without hyperglycemia. CST was the strongest independent risk factor for hyperglycemia in T2DM patients, whereas respiratory failure, reflected by the proportion of patients requiring invasive mechanical ventilation upon admission, was the strongest independent risk factor of hyperglycemia in non-diabetic patients. New-onset hyperglycemia was the most important risk factor for death in COVID-19 patients. The present study suggests that BG levels should be actively monitored in severe and critical COVID-19 patients. The occurrence of hyperglycemia in these patients, especially those without a previous diagnosis of T2DM, should be considered a marker of severity and worse outcomes. More studies are required to elucidate the likely multifactorial mechanisms of new-onset hyperglycemia in COVID-19 and the bidirectional interaction between new-onset hyperglycemia and severity in COVID-19 disease. We also need a prospective study investigating the impact of glycemic control in this group of patients and other similar diseases.
